# EVALUATING IMPLEMENTATION COSTS FOR A TRANSITIONAL PALLIATIVE CARE INTERVENTION TO SUPPORT RURAL CAREGIVERS

**DOI:** 10.1093/geroni/igac059.850

**Published:** 2022-12-20

**Authors:** Brystana Kaufman, Diane Holland, Catherine Vanderboom, Cory Ingram, Alice Chun, Erica Langan, Henry Baer-Benson, Joan Griffin

**Affiliations:** Duke University, Durham, North Carolina, United States; Mayo Clinic, Rochester, Minnesota, United States; Mayo Clinic, Rochester, Minnesota, United States; Mayo Clinic, Rochester, Minnesota, United States; Duke University, Durham, North Carolina, United States; Duke University, Durham, North Carolina, United States; Mayo Clinic, Rochester, Minnesota, United States; Mayo Clinic, Rochester, Minnesota, United States

## Abstract

Compared to urban caregivers, rural caregivers experience greater burdens accessing coordinated care for their loved ones during and after hospital discharge. The impact of technology-enhanced transitional palliative care (TPC) on patient and caregiver outcomes is currently being evaluated in a randomized control trial. This study evaluates resource use and health system costs of this caregiver-focused TPC intervention. Rural caregivers of hospitalized patients in Minnesota, Wisconsin, and Iowa were enrolled in an 8-week intervention consisting of video visits, conducted by a registered nurse, supplemented with phone calls and texts (n=207). Labor costs were estimated using the Bureau for Labor Statistics median hourly rate for a registered nurse and compared to a scenario analysis using a nurse practitioner or social worker wages. Hours spent conducting the visits and charting were calculated using study data. A one-way sensitivity analysis estimated resource use over a range of visits per caregiver and time per visit. Caregivers received 8.8 visits on average over the study period at 45 minutes per visit. In the base case, TPC cost $330 per caregiver facilitated by a registered nurse, compared to $281 and $489 if facilitated by a social worker or nurse practitioner, respectively. The number of visits had the greatest influence on total costs of the intervention (low of $198, high of $463). TPC is a feasible, low cost strategy to enhance caregiver support in rural areas. These results pose an opportunity to consider reimbursement mechanisms to evaluate the sustainability of transitional palliative care interventions to support caregivers.

